# Cardiometabolic risk in young adults from northern Mexico: Revisiting body mass index and waist-circumference as predictors

**DOI:** 10.1186/s12889-016-2896-1

**Published:** 2016-03-08

**Authors:** Abraham Wall-Medrano, Arnulfo Ramos-Jiménez, Rosa P. Hernandez-Torres, Rafael Villalobos-Molina, Diana C. Tapia-Pancardo, J. Rafael Jiménez-Flores, A. René Méndez-Cruz, Miguel Murguía-Romero, Itzell A. Gallardo-Ortíz, René Urquídez-Romero

**Affiliations:** Instituto de Ciencias Biomédicas, Universidad Autónoma de Ciudad Juárez, Anillo Envolvente del Pronaf y Estocolmo, Ciudad Juárez, 32300 Chihuahua México; Facultad de Ciencias de la Cultura Física, Universidad Autónoma de Chihuahua, Chihuahua, México; Unidad de Biomedicina, Universidad Nacional Autónoma de México, Tlalnepantla, México; Laboratorio Nacional en Salud: Diagnóstico Molecular y Efecto Ambiental en Enfermedades Crónico-Degenerativas, Facultad de Estudios Superiores Iztacala, Universidad Nacional Autónoma de México, Tlalnepantla, México

**Keywords:** Metabolic syndrome, Cardiometabolic risk, Body mass index, Waist circumference, Central obesity, Youden’s index

## Abstract

**Background:**

A body mass index (BMI) ≥30 kg/m^2^ and a waist circumference (WC) ≥80 cm in women (WCF) or ≥90 cm in men (WCM) are reference cardiometabolic risk markers (CMM) for Mexicans adults. However, their reliability to predict other CMM (index tests) in young Mexicans has not been studied in depth.

**Methods:**

A cross-sectional descriptive study evaluating several anthropometric, physiological and biochemical CMM from 295 young Mexicans was performed. Sensitivity (Se), specificity (Sp) and *Youden’s index* (*J*) of reference BMI/WC cutoffs toward other CMM (*n* = 14) were obtained and their most reliable cutoffs were further calculated at *Jmax*.

**Results:**

Prevalence, incidence and magnitude of most CMM increased along the BMI range (*p* < 0.01). BMI explained 81 % of WC’s variance [Se (97 %), Sp (71 %), *J* (68 %), *J*max (86 %), BMI = 30 kg/m^2^] and 4–50 % of other CMM. The five most prevalent (≥71 %) CMM in obese subjects were high WC, low HDL-C, and three insulin-related CMM [Fasting insulin, HOMA-IR, and QUICKI]. For a BMI = 30 kg/m^2^, *J* ranged from 16 % (HDL-C/LDL-C) to 68 % (WC), being moderately reliable (*J*max = 61–67) to predict high uric acid (UA), metabolic syndrome (MetS) and the hypertriglyceridemic-waist phenotype (HTGW). Corrected WCM/WCF were moderate-highly reliable (*J*max = 66–90) to predict HTGW, MetS, fasting glucose and UA. Most CMM were moderate-highly predicted at 27 ± 3 kg/m^2^ (CI 95 %, 25–28), 85 ± 5 cm (CI 95 %, 82–88) and 81 ± 6cm (CI 95 %, 75–87), for BMI, WCM and WCF, respectively.

**Conclusion:**

BMI and WC are good predictors of several CMM in the studied population, although at different cutoffs than current reference values.

## Background

Mexico’s epidemiological transition is advanced with a major burden coming from non-communicable chronic diseases. The fast urbanization, industrial development, technocratization and the apparent country’s prosperity, have improved the Mexicans lifestyle but simultaneously has diminished its health [1]. The rising adult mortality from cardiometabolic diseases (CMD), such as type 2 diabetes mellitus (T2DM) and hypertension (HTN) [2], a sudden (2014–2016) increase in their prevalence (8–12 %) and an unstoppable increment in treatment annual costs (US$/patient): 485 to 622 (HTN), 699 to 748 (T2DM) [3] are all indicative of the diminishing health state of the people. As if this were not enough, health inequities by region and population segments are still evident despite the government’s efforts to reduce them [4–6]. Therefore, it is increasingly urgent the timely diagnosis of CMD, by applying highly sensitive, reliable and economical methods, in order to reduce their burden for public health.

Obesity [body mass index (BMI) ≥30 kg/m^2^] and central adiposity [waist circumference (WC) ≥80 cm women (WCF), ≥90 cm men (WCM)] are prodromal conditions for many CMD [7, 8], including T2DM, metabolic syndrome (MetS) and cardiovascular disease (CVD). BMI and WC are more accurate than measured body fat by dual X-ray absorptiometry (DEXA), being WC a better predictor of these CMD and other inflammatory diseases [9, 10]. However, there are other CMD risk markers (CMM): HTN (≥130/≥85 mmHg), dyslipidemias [triacylglycerides (TAG ≥150 mg/dl), total cholesterol (TC ≥200 mg/dl), low-density lipoprotein cholesterol (LDL-C ≥100 mg/dl), and high-density lipoprotein cholesterol (HDL-C <50 mg/dl women, <40 mg/dl men)] and high fasting glucose (FG ≥100 mg/dl) and insulin (FI ≤14.0 μU/ml women, ≤11.0 μU/ml men [11, 12]). Clustered CMM includes the hypertriglyceridemic-waist phenotype (HTGW), atherogenic index (TC/HDL-C, >3.6 women, >4.3 men), TAG/HDL-C (>2.4 women, >2.9 men), insulin resistance (IR: HOMA-IR >2.9 women, > 2.3 men), and quantitative insulin sensitivity check index (QUICKI ≤0.33) [8, 13–15]. Also, since therapeutic lifestyle changes remain an essential modality in the clinical management of CMD, other health determinants including age (≥55 y woman, ≥45 y men), family history of CMD, smoking, sedentary lifestyle and high saturated fat intake, are also considered risk factors [16, 17].

International (e.g. WHO or NHBLI) and Mexican cutoffs for each CMM, including BMI and WC, have been established so far. However, most of them are influenced by ethnicity, gender, age and the presence of co-morbidities [18]. That is why, their sensitivity (Se) and specificity (Sp) changes from one population/region/country to another [19, 20]. As in case of age, according to the *2006 National Health & Nutrition Survey* [21] ~50 % of Mexican adults (≥20 y) had at least one altered CMM, 36.8 % have MetS [22], and the most common CMM were low HDL-C (76.8 %), high WC (73.6 %) and the triad HDL-C/HTN/WC (20 %) while high TAG (30.9 %) and the triad TAG/HTN/FG (0.5 %) ranked the lowest prevalent. According to this survey, age and location (urban/rural) have an impact on MetS prevalence but gender and socioeconomic status do not. However, ~72 % of young Mexicans (17–25 y) have at least one altered CMM, 13.4 % have MetS, and the most prevalent CMM were low HDL-C (41.8 %) and high WC (38.3 %) and TAG (18.5 %), and gender, age and even region modify MetS prevalence [8, 14, 19].

Although international standardization of CMM cutoffs allows the timely comparison and monitoring of health policies worldwide, the use of age-specific cutoffs to evaluate intervention programs in Mexico should be carefully selected [23–25]. The aim of this study was to evaluate the predictive value of BMI and WC toward other CMM abnormalities in young Mexicans, using the *Youden’s index* (*J*) for improving their specific cutoffs to achieve the highest Se and Sp (*J*max) [26, 27].

## Methods

### Study design & population

A cross-sectional descriptive study with a randomized sampling and multivariate (in BMI) and bivariate (in WC) stratification was performed. 2683 students (18.7 ± 2.7 y; age range 17–38) admitted in August 2014 by the Autonomous University of Ciudad Juarez (UACJ), were considered the initial universe. UACJ matriculates 3900 ± 500 students every six months, all considered participants in the “*Healthy University*” project, a comprehensive program that fosters academic performance by improving health and quality of life of students as well as their empowerment as public health promoters [28]. The sample size was considered large enough (11 %) to detect (5 % precision) the lowest CMM (high FG = 2 %) found in a preceding study [8] with a larger population (22 ± 5 y, *n* = 8144, 49 % women). However, as the purpose of the study was to validate BMI and WC as independent predictors of other CMM, a randomized sampling (based on the automatic selection of cases by school enrollment numbers) with a replacement strategy was performed in order to obtain proportional samples (50 % women/ 50 % men) within 4 BMI groups (*n* = 294, *n* = 71–77 per group) with the following demographics: 50 % female, 19 ± 2 y, medium-high socioeconomic level. All students perceived themselves as healthy and no CMD was diagnosed by the assigned and trained physician.

### Data collection procedure

*A) Anthropometry*: Height was determined to the nearest 0.1cm using a mobile stadiometer (SECA 208, Birmingham, UK), with the subject’s head in the Frankfurt plane. Body weight was determined to the nearest 100g using a digital scale (Tanita 682, Illinois, USA). Body mass index was then calculated [BMI = weight (kg) /height (m)^2^]. WC was measured to the nearest 0.1cm midway between the top of the ileac crest and the bottom of the rib cage, perpendicular to the trunk long axis, using a non-stretch measuring tape. These procedures were performed as recommended by the International Society for the Advancement of Kinanthropometry (ISAK) and the WHO STEPS protocol. *B) Blood pressure (BP):* It was measured with a manual aneroid sphygmomanometer (Model DS44, Welch-Allyn) to the nearest 1 mmHg in a seated position with the dominant arm resting and the palm facing upwards. The first Korotkoff sound marked the systolic (SBP) and the fifth the diastolic (DBP) blood pressure. Average readings (*n* = 3, every 5 min) were recorded at least 15 min after the participant arrival to the laboratory. *C) Biochemistry:* Students came to the University laboratory facilities between 7 and 10 AM after overnight fasting. Venous (antecubital) blood samples were obtained in suitable Vacutainer® tubes by trained biochemists responsible for sample collection, and all analyses were performed by Grupo Diagnóstico Médico PROA, S.A. de C.V., a Mexican internationally certified and accredited reference laboratory. Blood samples were centrifuged and the supernatant was recovered and used to measure the following biochemical parameters: FG, Uric acid (UA), TAG, CT, and HDL-C (mg/dL) by automatized enzymatic-colorimetric methods with an overall intra- and inter-assay coefficients of variation (CV) of <5 % and insulin (μU/ml) by ELISA; sensitivity was 0.5 μU/mL, and CV <5 %. Non HDL-C, LDL-C [29], HOMA-IR [16] and QUICKI [13], were then calculated with the following equations:

1 $$ \mathrm{N}\mathrm{o}\mathrm{n}\ \mathrm{H}\mathrm{D}\mathrm{L}-\mathrm{C} = \mathrm{T}\mathrm{C} - \left(\mathrm{H}\mathrm{D}\mathrm{L}-\mathrm{C}\right) $$

2 $$ \mathrm{L}\mathrm{D}\mathrm{L}-\mathrm{C} = \mathrm{T}\mathrm{C}\ \hbox{--}\ \left(\mathrm{H}\mathrm{D}\mathrm{L}\hbox{-} \mathrm{C}\right)\ \hbox{--}\ \mathrm{T}\mathrm{AG}/5 $$

3 $$ \mathrm{HOMA}-\mathrm{I}\mathrm{R} = \mathrm{F}\mathrm{I}*\mathrm{F}\mathrm{G}/405 $$

4 $$ \mathrm{QUICKI} = 1/\ \left[ \log\ \left(\mathrm{F}\mathrm{I}\right) + \log\ \left(\mathrm{F}\mathrm{G}\right)\right] $$

### Operational definitions

Students were classified at risk of CMD if they had at least one altered CMM, or as healthy if they had none of them. MetS was defined according to the consensus definition (IDF/NHLBI/AHA/WHF/IAS/IASO) [30] if they met at least 3 of the following traits or conditions: WC (WCF ≥80 cm, WCM ≥90 cm), TAG (≥150 mg/dl), HDL-C (<50 mg/dl women, <40 mg/dl men), HTN (≥130/≥85 mmHg) and FG (≥100mg/dl). Hyperuricemia (UA) was defined here as UA (mg/dl) >7.0 women, >8.0 men [31].

### Statistical analysis

**Fig. 1:**
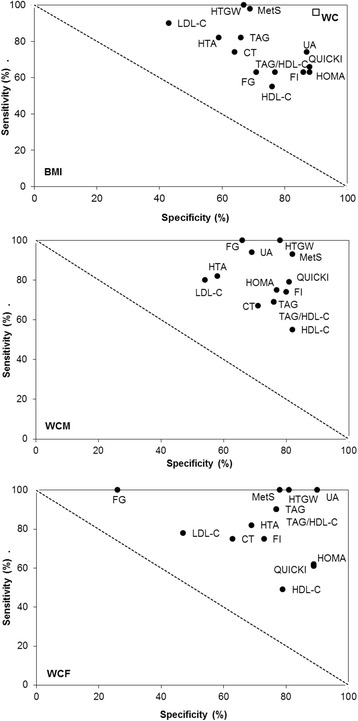
*J*max-corrected sensitivity & specificity of BMI and WC to predict other CMM. Legend: cardio-metabolic risk marker (CMM), see other abbreviations in text

All statistical analyses were performed with SPSS statistical software package version 22.0 (SPSS Inc., Chicago, IL, USA). All anthropometric, physiological and biochemical parameters were evaluated in stratified groups: A) By BMI [*n* = 71–77 each: <18.5 (underweight), 18.5–24.9 (normal), 25.0–29.9 (overweight) and ≥30.0 kg/m^2^ (obese)], B) In two (normal & high) WCF/WCM groups, Goodness-of-fit (R^2^) between BMI and other CMM was performed, adjusting data to the best regression curve possible (linear, quadratic, cubic, exponential or power). The reliability of reference standard BMI (25 and 30 kg/m^2^), WCF ≥80 cm and WCM ≥90 cm cutoffs as independent predictors of other CMM abnormalities, was analyzed by their sensitivity (Se, low false negatives) and specificity (Sp, low false positives) using crosstabs [14], receiver operating characteristic (ROC) curves, and *Youden’s Index* (*J*) [26]. *J* (Eq. 5) is defined at all points of a ROC-curve and its maximum value (*J*max = highest Se and Sp) is used as a criterion for selecting the optimum or most reliable cutoff (Fig. 1).

5 $$ J = \mathrm{Sensitivity}\ \left(\mathrm{S}\mathrm{e},\ \%\right) + \mathrm{Specificity}\ \left(\mathrm{S}\mathrm{p},\ \%\right)\ \hbox{--}\ 100 $$

## Results

CMM values (mean ± SD) increased across BMI categories (*p* ≤ 0.01, Table 1). WC, the 1st and 4th most prevalent CMM in obese and overweight subjects respectively (Table 2), showed the strongest linear (R^2^ = 0.69) or cubic (R^2^ = 0.81) relationship with BMI. FG (1–5 %), TC and UA (3–20 %) were the less prevalent CMM across BMI categories. Quadratic regression did not improve the linear trend between BMI and most CMM, while power (Log-Log) regression improved that of HOMA-IR, TC/HDL-C and TG/HDL-C. Except for WC (81 %), BMI explained 4 to 50 % of the associated variance to all other CMM, regardless of the goodness-of-fit model.

**Table 1:** Cardio-metabolic risk markers (CMM) stratified by BMI (kg/m^2^) categories

CMM^a^	<18.5	18.5 to < 24.9	25.0 to 29.9	≥ 30.0	R^2^		
					Linear	Other^b^	Type^b^
BMI	17.4 ± 0.8	21.8 ± 1.8	26.9 ± 1.4	34.9 ± 5.3			
WC	62.5 ± 4.8	70.7 ± 6.3	82.0 ± 7.5	96.5 ± 10.7	0.69	0.81	B
SBP	105.0 ± 11.0	110.0 ± 14.0	117.0 ± 11.0	120.0 ± 16.0	0.13	0.18	A
DBP	69.0 ± 9.0	72.0 ± 9.0	76.0 ± 11.0	79.0 ± 11.0	0.11	0.14	A
HDL-C	46.9 ± 8.7	45.2 ± 9.1	42.4 ± 8.9	37.9 ± 11.3	0.08	0.17	B
LDL-C	80.4 ± 21.1	98.2 ± 28.3	98.5 ± 25.2	111.2 ± 37.9	0.10	0.12	A
Non HDL-C	95.9 ± 22.8	115.3 ± 30.9	116.1 ± 29.4	138.0 ± 43.2	0.14	0.17	B
TC	142.8 ± 25.6	160.5 ± 31.1	158.6 ± 28.2	175.9 ± 41.5	0.09	0.11	D
TC/HDL-C	3.1 ± 0.6	3.7 ± 1.0	3.9 ± 1.0	5.1 ± 2.3	0.16	0.22	D
TAG	77.0 ± 24.0	87.5 ± 42.9	98.2 ± 37.8	144.9 ± 96.0	0.11	0.14	A
TAG/HDL-C	1.7 ± 0.7	2.1 ± 1.4	2.5 ± 1.3	4.9 ± 6.1	0.07	0.17	D
FG	77.1 ± 9.0	77.4 ± 8.1	81.2 ± 10.7	82.0 ± 12.2	0.04	0.50	A
FI	9.0 ± 7.7	10.0 ± 5.6	13.9 ± 12.3	20.8 ± 11.6	0.17	0.27	C
HOMA-IR	1.8 ± 1.6	2.0 ± 1.2	2.9 ± 2.9	4.3 ± 2.6	0.14	0.25	D
QUICKI	0.37 ± 0.03	0.36 ± .03	0.34 ± 0.03	0.32 ± 0.02	0.23	0.26	A
UA	4.4 ± 1.0	4.9 ± 1.0	5.3 ± 1.3	6.0 ± 1.5	0.12	0.20	B

Values are shown as mean ± SD. R^2^ at *p* < 0.01; ^a^See abbreviations and units in text; ^b^Quadratic (A), cubic (B), exponential (C), power (D) regression

**Table 2:** Prevalence (%) of altered CMM by BMI (kg/m^2^) category

CMM^a^	<18.5	18.5 to <24.9	25.0 to 29.9	≥30.0	Δ_N-OW_	Δ_OW-OB_
WC	0	1	39	92	38	53
HTA	7	8	28	39	20	11
HDL-C (↓)	44	42	66	71	24	5
LDL-C	13	47	41	58	6	11
TC	3	5	9	20	4	11
TAG	1	7	13	31	6	18
TAG/HDL-C	7	21	28	47	7	19
FG	1	1	3	5	2	2
FI (↑)	16	18	43	81	25	38
HOMA-IR	13	17	35	75	18	40
QUICKI (↓)	10	15	31	75	16	44
HTGW	0	0	9	30	9	21
UA	0	1	4	20	3	16
MetS	0	0	16	42	16	26

*P-trend* <0.01; ^a^See abbreviations and units in text; otherwise indicated, a higher CMM value (than cut off point) was considered altered. Increment (Δ) from normal-overweight (N-OW), from overweight-obesity (OW-OB)

Both, prevalence (Table 2) and incidence (Table 3) of abnormal CMM increased across BMI categories (*p* ≤ 0.01). However, increments (Δ) in lipid-related CMM, UA, FG and HTGW were low between normal to overweight (Δ_N-OW_). However, except for TC, HDL-C, LDL-C, HTA and FG (+2–11 %), huge increments (+16–53 %) in all other CMM were observed from overweight to obesity (Δ_OW-OB_). The five most prevalent (≥71 %) CMM in obese subjects were high WC, FI, HOMA-IR, QUICKI and HDL-C while the less prevalent was FG (5 %). An additional 21, 26 and 40 % subjects with HTGW, MetS and IR, respectively, increased from overweight to obesity. Also, 74–75 % under- or normal weight subjects had 1–2 altered CMM, 58 % overweight subjects had 3–6 altered CMM, and 54 % obese subjects had 6–8 altered CMM.

**Table 3:** Incidence (%) of altered CMM by BMI (kg/m^2^) category

#	<18.5	18.5 to <24.9	25.0 to 29.9	≥30.0
1	35.2	26.3	7	3.9
2	39.4	28.9	14.1	5.2
3	9.9	18.4	26.8	5.2
4	11.3	9.2	9.9	7.8
5	1.4	5.3	5.6	9.1
6	1.4	3.9	15.5	22.1
7	1.4	5.3	8.5	16.9
8		2.7	8.5	15.3
9			4.1	6.7
≥10				7.8

Number of altered CMM (#)

Reliability [sensitivity (Se, low false negatives) + specificity (Sp, low false positives)] of both BMI cutoffs (25 and 30 kg/m^2^) to predict WC changes did not vary much: [*Youden’s Index* (*J*), 68 to 74] reaching 97 % (Se) and 71 % (Sp) in obese subjects (Table 4). However, changing the BMI from 25 to 30 kg/m^2^ decreased Se but increased Sp to the same extent (~24 % in average) when predicting other CMM. With a BMI = 30 kg/m^2^, the lowest and highest Se was observed for HDL-C and LDL-C (33–34 %) and UA and HTGW (79 %), respectively while its Sp was for WC and FG (71–75 %) and HOMA-IR/FI/QUICKI (90–91 %). WCF showed practically the same prediction power like WCM (same Se and Sp) toward the same CMM: Lowest (HDL-C and LDL-C) and highest (UA, HTGW, MetS) Se, lowest (TC) and highest (FI, HOMA-IR, QUICKI) Sp. *J* ranged from 16 % (HDL-C and LDL-C) to 68 % (WC) for BMI (30 kg/m^2^), from 14 % (LDL-C) to 74 % (MetS) for WCF and from 23 % (LDL-C) to 78 % (HTGW) for WCM.

**Table 4:** Se, Sp and Youden’s Index (*J*) of BMI & WC to detect other CMM abnormalities

CMM^a,b^	BMI (kg/m^2^)						WC(cm)					
	25			30			WCF			WCM		
	Se	Sp	*J*	Se	Sp	*J*	Se	Sp	*J*	Se	Sp	*J*
WC	99	75	74	97	71	68						
HTA	82	58	40	49	80	29	71	69	40	57	78	35
HDL-C (↓)	62	65	27	33	83	16	43	81	24	52	82	34
LDL-C	62	58	20	34	82	16	45	69	14	45	78	23
TC	78	53	31	56	77	33	58	67	25	67	71	38
TAG	85	55	40	62	79	41	90	68	58	69	76	45
TAG/HDL-C	74	59	33	50	83	33	53	71	24	64	80	44
FG	75	51	26	50	75	25	75	64	39	75	68	43
FI (↑)	79	69	48	53	91	44	64	80	44	58	88	46
HOMA-IR	79	65	44	55	90	45	69	79	48	58	85	43
QUICKI (↓)	82	65	47	59	90	49	67	79	46	65	85	50
HTGW	100	55	55	79	80	59	100	69	69	100	78	78
UA	95	53	48	79	78	57	100	68	68	77	73	50
MetS	100	58	58	74	82	56	100	74	74	93	82	75

^a^Otherwise specified, a higher CMM value (than cut-off point) was considered at risk for CMD, sensitivity (Se, %), specificity (Sp, %). *J* = Se (%) + Sp (%) – 100 [25], ^b^See text for abbreviations and units

**Fig. 2:**
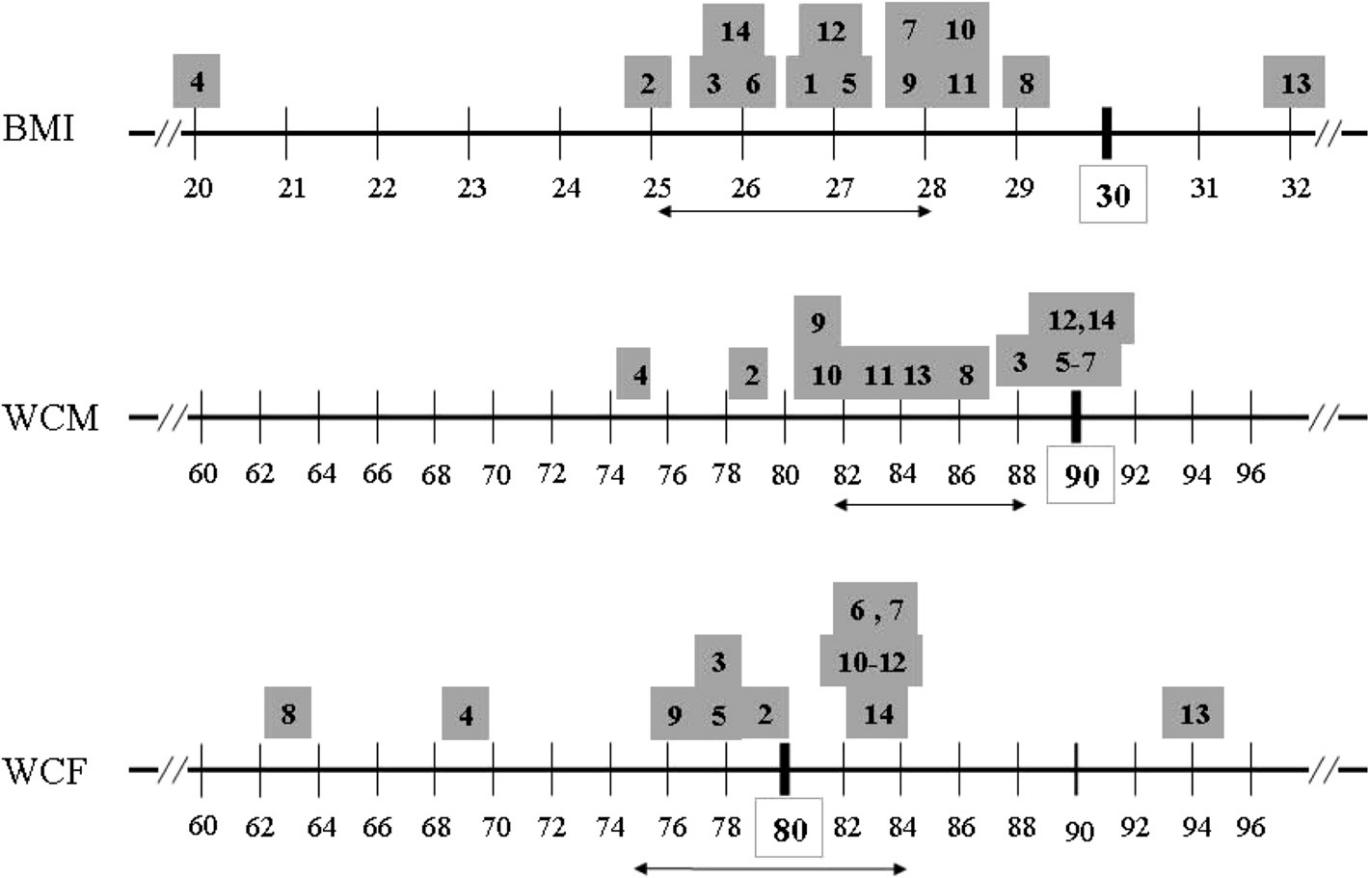
CMM distribution along BMI and WC range^1^. Legend: ^1^ *J*max –corrected cutoffs; Conventional BMI and WC (WCM/WCF) cutoff values (*bold*), *J*max-derived cutoff values to predict individual (*grey square*) and CI 95 % (↔) CMM; cardio-metabolic risk markers (CMM): WC (1), HTA (2), HDL-C (3), LDL-C (4), TC (5), TAG (6), TAG/HDL-C (7), FG (8), FI (9), HOMA-IR (10), QUICKI (11), HTGW (12), UA (13), MetS (14)

When plotting the most reliable Se & Sp (that is, those obtained at *Jmax*), it was possible to graphically identify the best predicted CMM by BMI (up), WCM (center) and WCF (down), as the closest CMM located at the upper right corner of each graph (Fig. 1). As expected, BMI was a strong predictor of WC (*J*max = 68–74 %) and to a lesser extent of HTGW, UA and MetS (*J*max = 48–59 %). WCM predicted in strong manner HTGW, MetS, UA and QUICKI (*J*max = 50–78 %) while WCF predicted MetS, HTGW, and UA (*J*max = 68–74 %). The less reliable prediction at *J*max was as follows: HDL-C & LDL-C, (*J*max = 14–34 %; BMI/WCF/WCM), TC (*J*max = 31–38 %, BMI/WCM/WCF), and FG (*J*max = 25–43 %, BMI/WCF/BMI). Lastly, specific BMI and WC cutoffs to predict other CMM with the highest reliability (at *J*max) are depicted in Fig. 1. Lastly, standard cutoffs for Mexican adults (**bold**) differed from those obtained at *J*max [specific (grey squares), CI95 % (**↔**)]: Distribution of most CMM was moderate-highly reliable at 27 ± 3 kg/m^2^ (CI 95 %, 25–28), 85 ± 5 cm (CI 95 %, 82–88) and 81 ± 6cm (CI 95 %, 75–87), for BMI, WCM and WCF, respectively (Fig. 2).

## Discussion

Overweight and obesity in young Mexicans has reached epidemic proportions. According to the *2012 National Health & Nutrition Survey* [32], four (17–19 y) and five (20–29 y) out of ten young Mexicans have a BMI ≥25 kg/m^2^, most of them bearing a morbid WC. BMI and WC have increased overtime in Mexicans [33] as a consequence of lifestyle changes (e.g. low physical activity and unhealthy dietary habits); however, as compared to BMI, WC has increased disproportionately from 1999 to 2012, particularly in young (20–29 y) woman (WCF: +6.6 cm, *p* < 0.0001) [34]. The *National Agreement for Nutritional Health* (*ANSA*) and other public policies aimed to prevent and control obesity, should be strengthened and improved urgently since there is no evidence to infer that this trend will decrease in the near future [1, 23, 33], particularly if intervention & surveillance programs are not properly designed, implemented and evaluated. About this, BMI and WC cutoffs established by international organizations (e.g. WHO or NHBLI) are often used to evaluate not only their secular trends but also as surrogate measures to evaluate CMD risk. However, BMI and WC cutoffs are influenced by ethnicity, gender, age and the presence of co-morbidities [9, 18, 35, 36], so age-specific cutoffs to evaluate intervention programs in Mexico should be carefully selected [23–25].

A BMI ≥25 kg/m^2^ is associated with greater odds of CMM abnormalities, MetS, IR, HTN, and T2DM [2, 11, 12]. That is why many of studies published in the last ten years have been focused not only on CMM’s epidemiology but also on the specific clustering patterns of BMI with other CMM [35, 36]. However, very few have explored the prevalence or incidence of CMM along the BMI range, particularly in Mexican youth [19]. This study shows that, with few exemptions (e.g. FG), the magnitude, prevalence and incidence of any CMM increase across BMI categories (*p* ≤ 0.01), with huge increments between 25 and 30 kg/m^2^. This behavior is supported by the fact that, in many cases, the mathematical relationship between BMI and each CMM improved with a nonlinear goodness-of-fit. The five most prevalent (≥71 %) CMM in obese subjects were high WC, HDL-C and three insulin-related CMM. Also, 16 % (overweighed) and 42 % (obese) subjects presented ≥3 MetS traits, and an additional 21, 26 and 40 % subjects with CMM-clustered phenotypes (HTGW, MetS and IR, respectively) were added from overweight to obesity. Based on these results, BMI is still valuable for the primary screening of CMD in school health programs such as *Healthy University* [28]. Many cross-sectional studies also support the usefulness of BMI for CMD screening [35–37], but also support the idea that BMI is an even better predictor if it is co-assayed or clustered with other anthropometric [e.g. WC, fat (FMI) and lean body mass index (LBMI)] or biochemical (e.g. such as TAG or HDL-C) CMM, either as simple risk scores (ratios) or in prospective equations. In our case, the study of novel associations between BMI (or WC) with the observed low prevalent (e.g. FG) or mild-BMI sensitive (e.g. TC, HDL-C) CMM, deserves further investigations.

However, despite the fact that 74–75 % subjects with a BMI <25 kg/m^2^ only had 1 or 2 altered CMM, ~46 % lean (18.5 to < 24.9 kg/m2) subjects were dyslipidemic (↑LDL-C, TAG/HDL-C; ↓HDL-C) while ~17 % were insulin-resistant (↑FI, ↑HOMA-IR). This “silent” phenomenon has several causes including the Mexican’s genetic predisposition to dyslipidemias [38] and T2DM [39], or the higher prevalence of snacking [40] and intake of junk food [41] that is quite popular in this population segment. However, these findings also indicate that weight gain itself do not impose a concurrent CMD risk in younger people from northern Mexico. In the OPUS PRIME study [35] the relative frequency of nine phenotypes [36 % lean, 43 % overweighed, and 21 % obese Mexican adults clustered in three CMM profiles (MetS traits): normo-metabolic (none), intermediate (1–2) and dysmetabolic (≥3)] was evaluated; the study showed that only 27 % lean subjects were normo-metabolic, while only 37 % of obese individuals were dysmetabolic. Murguía-Romero et al. [19] also reported from a national representative study with 3176 young (17–24 y) that all BMI strata included a MetS prevalence greater than zero (5.7 % lean, 21.8 % overweighed, and 48.6 % obese), implying once again that weight gain is not a mandatory factor for MetS.

WC is a crude estimate of abdominal fat accumulation and its measurement provides additional information on CMD risk at any given BMI value. As compared to BMI, WC is a better predictor of certain dysmetabolic processes such as inflammation, dyslipidemias and non-insulin dependent diabetes mellitus [8–11, 20]. Here, conventional WC cutoffs for Mexican adults (WCM/WCF) were moderate reliable (*J* = 69–78) to predict MetS and HTGW (both including a high WC in their definition) and UA in women (*J* = 68). In a previous study we have reported the excellent reliability of HTGW as surrogate measure for MetS in young Mexicans [8], since it is related to a higher weight, BMI, FG, TC, BP and to lower levels of HDL-C, which in turn are associated to a higher risk for HTN and IR. So, the binary association implied in HTGW (high TAG, high WC) normalizes WC to increase its Se and Sp to predict other CMM. Since young overweighed and obese individuals with a lower fat mass are more prone to IR than sex- and BMI-matched adults [42], the specific relationship between WC and truncal body fat distribution should be evaluated in future studies. Also, hyperuricemia (UA) is an independent factor associated with MetS and hypertrigliceridemia [31], HTA, IR, fatty liver and chronic kidney diseases [43]. To our knowledge, this is the first report on the association of WC with UA in young Mexicans.

However, WC had a very low predictive value toward lipid related-CMMs (*J* = 14–58). This could be related to several metabolic factors such as the high hemodynamics of cholesterol or the adipocyte’s TAG turnover rate, both influenced by age and BMI status [44–47], although it could also be related to the small number of participants in this study. Nevertheless, WC was the most prevalent CMM in obese subjects and BMI explained almost all (81 %) of its associated variance with an excellent reliability (*J*max = 86 %), which is consistent with other studies in young populations [48]. Under the caution that deserves the comparison due the small population sample, the prevalence of all CMM and their relationship with WC and BMI increments largely coincide with other cross-sectional studies in young Mexicans from central [8, 14] and northern Mexico [49].

Also, by using a very simple statistical tool (Youden’s index, *J*), the most reliable (*J*max) cutoff for BMI, WCM and WCF to predict other CMM was obtained. Other studies have also documented the increase in the reliability of several obesity indexes to predict cardiometabolic risk in pediatric populations, when their standard cutoffs have been corrected at *J*max [37, 50]. Here, most CMM abnormalities started at a lower BMI (27 ± 3 kg/m^2^) and WCM (80 ± 8 cm) but almost same the WCF (85 ± 5cm) than those proposed by WHO, as previously found in the 2000 (ENSA) and 2006 (ENSANUT) Mexican health surveys [25]. Strikingly, certain CMM reach their highest prediction at BMI and WC cutoffs out of their CI 95 % range (e.g. UA at WCF = 94 cm). However, it should be noted that enhancing Se and Sp to achieve *J*max of any CMM increases its likelihood ratio and odds ratio but do not minimize the epidemiological value of conventional cutoffs, it just informs about the possible “lost cases”.

## Stregths & limitations

The authors recognize that this study has certain strengths and limitations. The experimental design and sampling strategy allowed a reasonable comparison (same “n”) of the effects of weight gain and central obesity and several metabolic derangements (CMM) in a same study, and even when not intended to make inferences about the original universe, the behavior of this sample was quite similar to our previous study [8]. In addition, the assessment of certain CMM not previously reported in other studies of the same nature (e.g. UA), further contributes to the generation of knowledge in this field. However, its cross-sectional nature does not allow inferences on the current relationship of any CMM with a future risk for CMD. In addition, the sample size could result in no statistical significance for certain CMM with expected biological deviations. Nevertheless, our study could be considered as a “pilot” study, contributing to the design of more extensive cross-sectional or longitudinal studies.

## Conclusions

BMI and WC are good predictors of several CMM in the studied population, although at different cutoffs than current reference values. This study enables us to argue on the importance of the systematic measurement of WC and BMI, and possibly TAG, to follow the secular changes in CMD risk among college students.

## Ethics & consent statement

All participants signed an informed consent after explaining the nature, objectives and risks inherent to the study (when the participant was under 18 y, its parent/guardian signed the informed consent and the participant accepted to participate). Data confidentiality was protected as stipulated by the Mexican Statistical and Geographical information Law, and the study protocol met the standards of the Helsinki Declaration and was approved by UACJ ethics committee [8].

## Abbreviations

ANSA: National agreement for nutritional health
BMI: body mass index
BP: blood pressure
CMD: cardiometabolic disease
CMM: cardiometabolic risk marker
CVD: cardiovascular disease
DBP: diastolic BP
DEXA: dual x-ray absortiometry
FG: fasting glucose
FI: fasting insulin
HDL-C: high-density lipoprotein cholesterol
HOMA: homeostatic model assessment
HTA: hypertension
HTGW: hypertriglyceridemic-high waist phenotype
IR: insulin resistance
ISAK: International Society for the advancement of kinanthropometry
*J, Jmax*: Youden index
LDL-C: low-density lipoprotein cholesterol
MetS: metabolic syndrome
OB: obesity
OW: overweight
QUICKI: quantitative insulin check index
SBP: systolic BP
Se: sensitivity
Sp: specificity
T2DM: type 2 diabetes mellitus
TAG: triacylglycerides
TC: total cholesterol
UA: high uric acid
WC: waist circumference
WCF: waist circumference of female
WCM: waist circumference of male
